# Is Scientific Medical Literature Related to Endometriosis Treatment Evidence-Based? A Systematic Review on Methodological Quality of Randomized Clinical Trials

**DOI:** 10.3390/medicina55070372

**Published:** 2019-07-15

**Authors:** Roxana-Denisa Capraş, Andrada Elena Urda-Cîmpean, Sorana D. Bolboacă

**Affiliations:** 1 Department of Medical Informatics and Biostatistics, Faculty of Medicine, “Iuliu Hațieganu” University of Medicine and Pharmacy, 400349 Cluj-Napoca, Romania; 2 Department of Anatomy and Embryology, Faculty of Medicine, “Iuliu Hațieganu” University of Medicine and Pharmacy, 400006 Cluj-Napoca, Romania; 3 “Dominic Stanca” Gynaecology Clinic, 400124 Cluj-Napoca, Romania

**Keywords:** randomized clinical trial, endometriosis therapy, reporting quality, hierarchy of evidence, study replication

## Abstract

*Background and objectives:* Informed decision-making requires the ability to identify and integrate high-quality scientific evidence in daily practice. We aimed to assess whether randomized controlled trials (RCTs) on endometriosis therapy follow methodological criteria corresponding to the RCTs’ specific level in the hierarchy of evidence in such details to allow the reproduction and replication of the study. *Materials and Methods:* Using the keywords “therapy” and “endometriosis” and “efficacy” three bibliographic databases were searched for English written scientific articles published from 1 January 2008 to 3 March 2018. Only the randomized clinical trials (RCTs) were evaluated in terms of whether they provided the appropriate level of scientific evidence, equivalent to level 1, degree 1b in the hierarchy of evidence. A list of criteria to ensure study replication and reproduction, considering CONSORT guideline and MECIR standards, was developed and used to evaluate RCTs’ methodological soundness, and scores were granted. Three types of bias, namely selection bias (random sequence generation and allocation concealment), detection bias (blinding of outcome assessment), and attrition bias (incomplete outcome data) were also evaluated. *Results:* We found 387 articles on endometriosis therapy, of which 38 were RCTs: 30 double-blinded RCTs and 8 open-label RCTs. No article achieved the maximum score according to the evaluated methodological criteria. Even though 73.3% of the double-blinded RCTs had clear title, abstract, introduction, and objectives, only 13.3% provided precise information regarding experimental design and randomization, and also showed a low risk of bias. The blinding method was poorly reported in 43.3% of the double-blinded RCTs, while allocation concealment and random sequence generation were inadequate in 33.3% of them. *Conclusions:* None of the evaluated RCTs met all the methodological criteria, none had only a low risk of bias and provided sufficient details on methods and randomization to allow for the reproduction and replication of the study. Consequently, the appropriate level of scientific evidence (level 1, degree 1b) could not be granted. On endometriosis therapy, this study evaluated the quality of reporting in RCTs and not the quality of how the studies were performed.

## 1. Introduction

Informed decision-making requires that healthcare specialists possess the ability to identify and integrate evidence resulting from valid medical research. The dilemma is: how can a physician identify the appropriate scientific evidence, given the questionable integrity of published studies and their inability to answer the questions that truly matter in handling a specific patient? Too many research studies published in the scientific literature are improperly designed or provide insufficient details, thus hindering the reproduction of the study and the confirmation of the findings. Sometimes the published research can be deceptive, at least to some extent, influencing the practical implementation and use of the research results [1].

The quality of any scientific evidence can be affected by several issues, such as various academic interests and inappropriate advertising [2], the lack of transparency and independence of research projects (leading to failure of complying with the research protocol or to halt the research too soon) [3,4], ghost or guest authors (whose participation in the study is unclear) [5], the intentional concealment of bias [6], fraud [7,8], errors [9], and over-interpreted and over-evaluated results [10]. The readers and health policy-makers must consider all of these issues. Weak evidence leads to unsuitable medical decisions with direct consequences on the patient’s health care and quality of life.

Randomized clinical trials (RCTs) are ranked among the highest levels, second place, in the hierarchy of scientific evidence for research articles [11,12]. The RCTs are rigorous systematic approaches that answer specific clinical questions, rely on a large group of participants, and assure a low probability of bias by using a rigorous methodologic process [12,13,14]. Moreover, randomized clinical trials are the input of systematic reviews and meta-analyses; this type of secondary research approach makes it possible to enhance the clinical evidence and the creation of clinical guidelines [15,16,17,18]. Hence, the systematic reviews and meta-analysis outputs’ quality reflects the quality of RCTs used.

The quality of the methodology and reporting of a RCT is directly affected by the applied design, which is reflected in potential for the RCT’s results to be applied in clinical practice and in the results’ feasibility as input data for systematic reviews and meta-analyses. The “Consolidated Standards of Reporting Trials” (CONSORT) is a structured instrument developed to assist researchers in the reporting of clinical trials [18,19].

Endometriosis is a common disease in gynecology, marked by the presence of endometrial glands and stroma outside their location [20]. Although women with endometriosis can be asymptomatic, the symptoms are complex and vary from patient to patient, including chronic pelvic pain (most frequently), dysmenorrhea, dysuria, painful defecation, non-cyclical pain, and infertility [20,21]. The treatment of endometriosis is difficult, and the results are questionable. The therapeutic approach depends on the woman’s specific symptoms, their severity, the location of endometriosis lesions, the purpose of treatment and the wish to preserve fertility. It is essential that the evidence related to the treatment of endometriosis be high-quality, veridical, and well-documented [20].

In this study, randomized clinical trials reporting treatment of endometriosis were identified and critically evaluated according to a series of methodological criteria necessary in reproducing and replicating the study and to check the extent to which they fit into the RCT appropriate level of scientific evidence, level 1, degree 1b in the hierarchy of evidence.

## 2. Materials and Methods

### 2.1. Data Extraction

A search, using as keywords “therapy” and “endometriosis” and “efficacy”, was conducted on two databases developed by the US National Library of Medicine, National Institute of Health (namely PubMed and PMC) and on the ScienceDirect database. The search was done on the 3 March 2018 without restrictions regarding the type of manuscripts. For each English written article published between 1 January 2008 and 3 March 2018 (the date of search), its link was collected and stored. The articles reporting treatment of endometriosis regardless of the experimental design conducted on human subjects, were eligible for inclusion. The eligible articles were screened in two steps. In the first step, the title and abstract of the articles were screened, article duplicates and articles not related to endometriosis treatment were excluded. In the second step, the full text of the pool of articles resulted from the first step was screened. According to the information reported in the full-text, each article was classified according to study type as: randomized clinical trials, meta-analyses, systematic reviews, expert-witness case studies, cohort studies, case presentations, series of cases, in vitro studies, or studies on animals. Randomized clinical trials (RCTs) are the gold standard design for therapy interventions, classified as level 1, grade 1b in the hierarchy of evidence and designated as the most informative primary research whenever effectiveness is of interest [22]. For this reason, we conducted the evaluation of the methodological quality only on the RCTs reporting therapy of endometriosis, regardless of the type. 

### 2.2. Methods

The randomized clinical trials were divided into double-blinded randomized clinical trials (DB-RCTs) and open-label randomized clinical trials (OL-RCTs). The methodological quality of the RCTs was evaluated based on the criteria defined in Table 1. The criteria were constructed following methodological criteria from the CONSORT guideline [23], as well as a check-list for methodological assessment of randomized controlled trials [24]. Data abstraction, according to each criterion presented in Table 1, was collected and stored for each article included in the study. 

In the case of RD-RCTs, 1 point was assigned for each criterion established in Table 1 if that criterion was met; otherwise, zero points were granted. A total score of 26 points was the maximum an article could obtain for successfully accomplishing all the evaluated criteria, with 21 points being awarded for the details needed to replicate and reproduce the study being included (methods section). Regarding the OL-RCTs, a total score of 23 points was the maximum it was possible to obtain, with 18 points being for the methods section. Excellent quality of RCTs’ methodology, ensuring sufficient detail to enable the reproduction and replication of the study, was assumed when the maximum number of points was obtained in both the methods and randomization sections.

The risk of bias based on the following four categories, namely random sequence generation and allocation concealment (regarded as selection bias), blinding of outcome assessment (detection bias), and incomplete outcome data (attrition bias), was further evaluated for DB-RCTs. For each category, the bias was marked as green for low risk of bias, red for high risk of bias or yellow for unclear risk of bias. 

The appropriate level of valid scientific evidence for RCTs is level 1, degree 1b, according to the hierarchy of evidence for therapeutic studies (Oxford Centre for Evidence-Based Medicine, 2009). In our analysis, we considered as equivalent to level 1 degree 1b only those RCTs having a maximum score based on criteria provided in Table 1 and a low risk of bias in all four evaluated categories.

Next, the articles included in our evaluation were classified based on their publication in Web of Science (WoS) indexed journals or not. In the case of those published in journals indexed by WoS, the journal rank was also retrieved (Q1 as first quartile, Q2 as second quartile, Q3 as third quartile, and Q4 as fourth quartile; where Q1 is the highest rank), based on the year when the article was published.

### 2.3. Data Analysis

Data analysis was conducted mainly at the level of description, numbers and percentages were reported for qualitative data. The scores and the risk of bias were presented using either tables or graphical representations. Kruskal-Wallis ANOVA test, at a significance level of 5%, was used to compare the scores obtained according to the described method between articles published in WoS-Q1, WoS-Q2, WoS-Q3, WoS-Q4, and no-Q sub-groups, whenever the sample size allowed the comparison. The Statistica program (v. 8, StatSoft, Tulsa, OK, USA) was used for statistical analysis.

## 3. Results

### 3.1. Description of Study Retrieval

**Figure 1:**
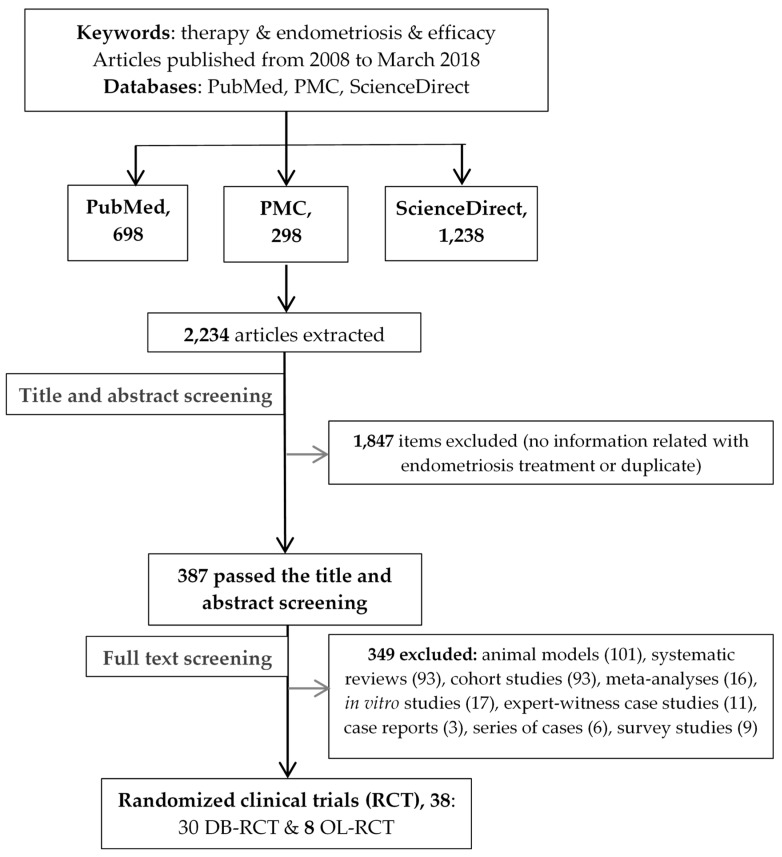
Flow diagram showing the process from searching the articles to title-abstract and full-text screening. (DB-RCT = double-blinded randomized clinical trial, OL-RCT = open-label randomized clinical trial).

The search strategy identified 2234 potentially relevant articles for the study. Most of the articles (1847) were excluded after title and abstract screening, since they did not present results related to endometriosis treatment. Almost four hundred articles entered full-text screening, and 38 were included in the final analysis. The reasons for exclusion are provided in Figure 1.

### 3.2. Analysis of Double-Blinded Randomized Clinical Trials

None of the evaluated DB-RCTs achieved the maximum total score of 26 points (scores in Table 2).

Only 73.3% (22/30) of the DB-RCTs included in the analysis had a maximum score of 4 for TABO sections (title/abstract/state of the art and objectives). 

In the Methods section, none of the DB-RCTs achieved the maximum of 17 points, 20% (6/30) of them had 16 points, and 30% (9/30) of them had 15 points (Table 2). Half (50%) of the DB-RCTs did not mention the treatment concealment method. The type of trial (i.e., parallel/factorial) was not mentioned in 66.7% (20/30) of the cases. Furthermore, in 43.3% (13/30) of the DB-RCTs, it was unclear who was blinded, the individuals subjected to treatment, the investigators, or both. Details about the method used to generate the random allocation sequence, on the mechanism used to implement the random allocation sequence, about who generated the random allocation sequence, who enrolled the participants and who assigned the participants to interventions were missing in one-third (10/30) of the evaluated DB-RCTs.

**Figure 2:**
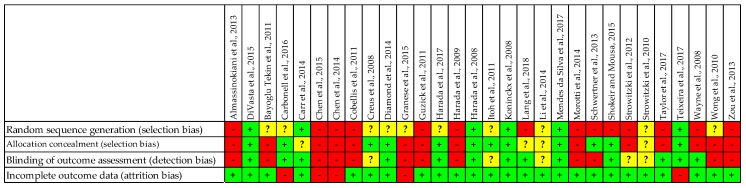
The evaluation of risk of bias for double-blinded randomized clinical trials. (“+” (green) = low risk of bias; “-” (red) = high risk of bias; “?” (yellow) = unclear risk of bias).

**Figure 3:**
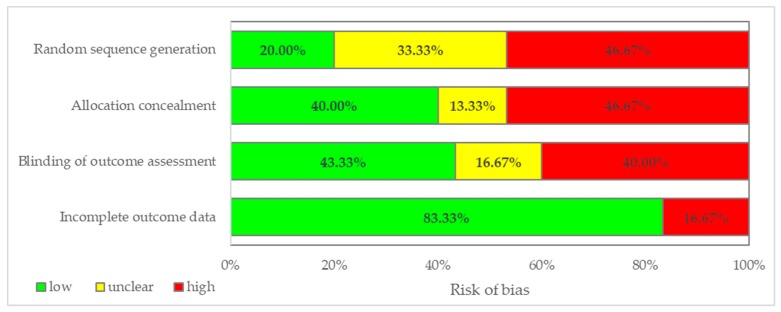
The distribution of the risk of bias for double-blinded randomized clinical trials.

High risk of bias was observed in 40% (12/30) DB-RCTs for both random sequence generation and allocation concealment (regarded as selection bias, Figure 2). Eighty percent of evaluated DB-RCTs presented a high or unclear risk of bias regarding random sequence generation (Figure 3). Furthermore, more than half of the evaluated articles had a high or unclear risk of bias with regard to allocation concealment (60%) and blinding of outcome assessment (56.7%). A small percent of evaluated DB-RCTs had a high risk for incomplete outcome data (16.67%, Figure 3).

Most of the DB-RCTs were published in journals indexed by WoS (28/30), the majority belonging to journals ranked in Q2 (9/28) or Q3 (9/28) and the minority belonging to those in the extreme quartiles (6/28 in Q1, 4/28 in Q4). No significant differences in scores were observed based on the rank of the journal where the article was published (including also the No-Q articles; Kruskal-Wallis ANOVA test: *p*-value = 0.47).

### 3.3. Analysis of Open-Label Randomized Clinical Trials

None of the eight OL-RCTs included in the study obtained the maximum total score of 23 points. Even though all OL-RCT had a structured abstract, only 75% (6/8) obtained a maximum score of four in TABO sections (Table 3). 

In the Methods section, just one article provided information for all evaluated items and achieved the maximum score (15), while 50% of OL-RCT cumulated 11 points. The score for randomization varied from zero to two, with none of the investigated articles achieving the full possible score (Table 3).

All the OL-RCTs included in the study were published in journals indexed by WoS, 37.5% (3/8) of them were published in Q4-ranked journals.

## 4. Discussion

The evaluated RCTs reporting on endometriosis treatments did not achieve either the maximum possible score in the presentation of the methods used and randomization applied, or a low risk of bias in each of the four categories. Thus, none of the evaluated RCT provided sufficient detail to allow the reproduction and replication of the experiment, and none could be assigned level 1, degree 1b in the hierarchy of evidence for therapeutic studies.

None of the evaluated DB-RCT achieved the maximum score of 26 points, but some had good scores on the presentation of the methods and randomization (Table 2). To be more precise, 73.3% of the 30 DB-RCTs got a maximum score in the TABO section, which is indicative of a clear title, a structured abstract, and precise objectives. No article reached the maximum of 17 points for the Methods section, but 50% got scores of 15 and 16 points. Almost half of the evaluated DB-RCTs did not provide the method of concealment of the treatment administered, nor did they reveal who had been blinded during the administration of the treatment (Table 2). Furthermore, the randomization section had the lowest scores: 60% (18/30) of the articles had 0 or 1 point, just 13.3% had accurate information about randomization reaching the maximum score (Table 2). The lack of complete transparency and completeness in the reporting of methods, data and analysis was observed in the evaluated studies; therefore, the knowledge must be carefully interpreted.

Only seven articles [26,34,37,39,41,44,51] gained an overall score ≥23 points (approximatively 88% of the maximum possible total score). Thus, they were the closest to reaching a high-quality status according to the evaluated methodological items. In addition, just four articles out of 30 were classified as having a low risk of bias in all four evaluated categories. Overlapping this result with the one above, we observed that the four articles with low risk of bias were also found in the list with the highest total scores [26,39,41,44] (Table 2 and Figure 2), thus being the most reliable of all 30 DB-RCTs. Since the presence of one or two unclear risks of bias could jeopardize the study’s validity, more details could be needed to interpret the reported findings properly. High risk of bias was observed among the evaluated DB-RCTs more frequently in the selection (46.7%), then in detection (40%), and finally in attrition (16.7%) (Figure 3).

The DB-RCTs had been published in journals indexed by Web of Science, with two exceptions. Publishing articles in journals considered influential, such as those indexed by WoS, leads to certain advantages for authors, such as greater prospects of employment or obtaining an academic position [63]. According to the WoS journal ranking based on impact factors, 18 out of 28 DB-RCTs were published in Q2 or Q3 journals. There were no significant differences between the total scores of articles published in journals regarding their ranks (Q1–Q4; Kruskal-Wallis ANOVA test: *p*-value > 0.4). However, none of the evaluate DB-RCTs gained maximum score in order to consider them of high quality, regardless of the journal that published them.

Similar to the results obtained by DB-RCTs, none of the OL-RCTs have achieved the maximum score of 23 points (Table 3). Only one OL-RCT achieved a score of 21 points (>90% of the methodological criteria provided in Table 1). Of the eight evaluated OL-RCTs, 75% got a maximum of 4 points in TABO section, indicative of a clear title, a structured abstract and precise objectives. In the Methods section, one article reached a maximum of 15 points, while 50% got scores of 11 points. No article reached a maximum of 3 points in randomization, six articles having 0 or 1 point regarding the accuracy of randomization information. In the Results section, all OL-RCTs presented the required information according to the evaluated criterion, receiving the maximum score of 1 point (Table 3). 

Controlled randomized studies were seen as better evidence, compared to observational, cohort-based, or expert-witness case ones, due to the low risk of bias managed through a rigorous design of experiment [64,65]. However, as Redwine et al. already reported [65], the concept of evidence-based medicine rather focused on the type of a study not on its quality, overestimating the randomized clinical trials and the evidence produced by them. Our study’s results point towards the same idea on the subject of endometriosis treatment.

The evaluation of the quality of randomized clinical trials in different medical fields were previously reported (Table 4), and all studies pointed out the lack of a rigorous presentation of the experimental design or different levels of possible biases.

Describing the elements needed for the execution of the study, collection of experimental data and analysis of data is necessary in order to allow reproduction (the same experimental design applied on a sample extracted from the same population) and replication (the same experimental design applied to a different population) of a study. The reproduction and replication of a study conducted on humans could validate the results, and thus translation of the research results in the current practice [15,16,17,18]. The experimental design of a RCT should offer sufficient details to allow reproduction and replication of the study and to verify the accuracy of the research results, before implementing them in daily clinical practice. Even though none of the evaluated RCTs in our study achieved a maximum score in the experimental design section, a substantial proportion of the evaluated articles provided a good description as compared to the RCTs summarized in Table 4. The results reported in our study, on the subject of endometriosis treatment, were better compared to those of other previous studies, showing an increase in the quality of reporting RCTs. The request of the publishers to report RCTs using the CONSORT guidelines or the training of researchers in reporting RCT research results could explain this result. 

A detailed presentation of the randomization, the creation and concealment of allocation sequence should be thoroughly described in the method section. More precisely, presenting the applied technique, explaining why a particular randomization technique was chosen, how the allocation sequence was generated, how the randomness was guaranteed, how the stratified randomization was applied and which prognostic variables were used (if applicable) are needed to ensure a successful reproduction and replication of a RCT [70]. Phrases such as “patients were randomly allocated to the treatment and control arm” should be avoided, because it lacks the details needed to reproduce and replicate the design of the experiment.

### Study Limitations and Recommendations for Future Research 

Our study is limited by the literature search conducted on only three databases (PubMed, PMC, and ScienceDirect), using only English written articles published until February 2018. A more extensive search, including other databases, articles written in other languages, and evaluation of the papers published since March 2018 (36 new eligible items published from March 2018 to July 10, 2019 have been indexed in PubMed) could report more accurately on the design quality in the RCTs on endometriosis treatment. Furthermore, extending the search to the grey literature and unpublished data could offer more insight into the evidence regarding endometriosis treatments, decreasing the effect of the publication bias. 

The criteria used to evaluate the reproducibility and replication of the studies reporting treatment for endometriosis were decided based on what the authors considered to be the most important information for study reproduction and replication. However, the research in medicine is growing very fast and other criteria could be considered as necessary in the evaluation of proper reporting of the research design, which allows for the reproduction and replication of a study. Furthermore, the applied scoring system considers the same weight for all investigated items. Several items could be considered more important for study reproduction and replication, so the development of a weighted score could reflect more accurate the completeness of the experimental design.

Our study aimed to evaluate the reproducibility and replicability of the study reporting an endometriosis treatment, so to perform a meta-analysis was out of our scope. Furthermore, the identified studies were heterogeneous with regard to the drug (the highest number of studies evaluating the same treatment—Dienogest—is six, four DB-RCTs [38,42,48,49] and two OL-RCTs [60,61]) and the used doses. Race/ethnic differences were observed with regard to both prevalence and symptoms [71,72,73] and therapy response [74,75], so it is recommended to carefully select the studies to be combined in the results of a meta-analysis. Several meta-analyses have already been published in the scientific literature, reporting the efficacy of Levonorgestrel releasing intrauterine system as post-operative therapy [76], the effect of micronized Palmitoylethanolamide-trans-polydatin on endometriosis-related pain [77], the comparison of Dienogest (DNG) with gonadotropin-releasing hormone (GnRH) analogs [74]. However, the effect of race/ethnicity on the results of meta-analyses, as well as the effects of different levels of bias also needs to be assessed. 

The limited number of articles included in this study is another limitation. Endometriosis is a condition with as-yet-unknown origin and pathogenesis and heterogenic symptoms not necessarily correlated with the extent of the disease [78,79]. The heterogeneity of patients’ characteristics imposes particular treatment from patient to patient and this is closely reflected in the small number of RCTs. In this regard, the quality assessment of all types of therapy interventions on endometriosis are needed, and such evaluations could add value and better reflect the transparency and completeness of the research methods relating the therapy of endometriosis.

A particular research consideration is required for endometriosis due to its association with increased risk of cardiovascular diseases [80,81], autoimmune diseases [82], and cutaneous melanoma, breast, ovarian, endometrial and cervical cancer [83]. Furthermore, the burden of this disease on individual women, their families and society [20] also support the needs for the elucidation of etiology and pathogenesis, early diagnosis considering the absence of symptoms and innovations for personalized therapy. 

Given that the evidence produced by scientific medical writing affects the ways patients are treated, the reproduction and/or replication of a reported intervention are a must in supporting the verification of the findings before their application in clinical practice. Insufficient details in the Methods section of a published RCT does not reflect the invalidity of the study, it shows at least poor reporting skills on behalf of the authors. Efforts are done to reflect also the quality not just the type of an experimental design (e.g., GRADE [84,85]) but space for improvement still exists [86]. Improving researchers’ skills in better reporting interventional research could permit an increase in the numbers of replicable (and thus verifiable) studies and a better assessment of the risk of bias. The quality of studies on humans is directly related to the available research infrastructure, research networks (allowing multi-center studies), high-quality researchers (e.g., skills and scientific behavior regarding the responsible research practice) and clear rules and regulations regarding the conduct of research, as well as high quality in reporting results. A joint continuous effort of academics and professional associations is needed to improve the quality of published scientific literature and to increase the transparency and completeness details regarding what and how research was done, thus ensuring the replication and reproduction of a study with direct implications for daily clinical practice. 

## 5. Conclusions

The score reflecting the description of the experimental design on the evaluated RCTs endometriosis therapy was generally high, but unfortunately, none of the articles could be considered to have sufficient detail to allow for the replication and reproduction of the study. A RCT can only be considered as level 1, grade 1b in the hierarchy of evidence when sufficient methodological details are provided. Our evaluation strictly reflects the reporting of the RCTs, but not how the RCTs were conducted, considering the proposed criteria and scoring system. On the subject of endometriosis treatment, even if RCTs presented a low risk of bias, their lack of details in the research design made study replication and reproduction problematic. Hence, verification of validity and reliability of the reported treatment could not be attained. 

## Figures and Tables

**Table 1:** Quality criteria used to evaluate randomized clinical trials reporting endometriosis treatment (OL-RCT = open-label randomized clinical trial).

Section	Checklist Item (Yes = 1 vs. No/Cannot Say = 0)
Title	Identification as a randomized trial in the title?
Abstract	2. Is the abstract structured? (Introduction/Methods/Results/Conclusion(s))
Background and objectives	3. Does the study address an appropriate and clearly focused question? 4. Are the objectives and hypotheses specified?
Methods	Is the assignment of subjects to treatment groups randomized? Is an adequate concealment method used? (except for OL-RCT) Description of trial design (such as parallel, factorial) Is the allocation ratio reported? Do eligibility criteria for participants exist? Are there enough eligibility criteria? Settings and locations were reported? Do the interventions for each group offer sufficient details to allow replication, including how and when they were actually administered? Does the design keep subjects and investigators ‘blind’ about treatment allocation? (except for OL-RCT) Are the treatment and control groups similar at the start of the trial? Is the treatment under investigation the only difference between the groups? All relevant outcomes are measured in a standard, valid, and reliable way? Is the primary outcome specified? Are the secondary outcomes specified? Did any changes to the trial outcomes occur after the trial for a good reason? Is the information regarding the number of individuals or clusters recruited into the treatment arms of the study that dropped out before the end of the study reported? Are all the subjects analyzed in the groups to which they were randomly allocated (often referred to as intention to treat analysis)?
Randomization	The method used to generate the random allocation sequence The mechanism used to implement the random allocation sequence (such as sequentially numbered containers) Who generated the random allocation sequence, who enrolled participants, and who assigned participants to interventions If done, who was blinded after assignment to interventions (except for OL-RCT)
Results	Are the results directly applicable to the patient group targeted?

**Table 2:** The scores for DB-RCTs (TABO = Title/Abstract/Background and Objectives; Max. score = 26 pt.).

Author, Year [Ref]	TABO	Methods	Randomization	Results	Total Score
Almassinokiani et al., 2013 [25]	3	10	0	1	14
DiVasta et al., 2015 [26]	4	15	4	1	24
Bayoglu Tekin et al., 2011 [27]	4	12	1	1	18
Carbonell et al., 2016 [28]	4	12	3	1	20
Carr et al., 2014 [29]	3	16	2	1	22
Chen et al., 2015 [30]	4	12	0	1	17
Chen et al., 2014 [31]	4	10	0	1	15
Cobellis et al., 2011 [32]	3	12	0	1	16
Creus et al., 2008 [33]	4	15	2	1	22
Diamond et al., 2014 [34]	3	16	3	1	23
Granese et al., 2015 [35]	4	13	1	1	19
Guzick et al., 2011 [36]	4	12	0	1	17
Harada et al., 2017 [37]	4	16	3	0	23
Harada et al., 2009 [38]	4	14	0	1	19
Harada et al., 2008 [39]	4	16	4	1	25
Itoh et al., 2011 [40]	3	16	2	1	22
Koninckx et al., 2008 [41]	4	16	4	1	25
Lang et al., 2018 [42]	4	15	1	1	21
Li et al., 2014 [43]	4	15	1	1	21
Mendes da Silva et al., 2017 [44]	4	14	4	1	23
Morotti et al., 2014 [45]	3	12	0	1	16
Schwertner et al., 2013 [46]	3	15	1	1	20
Shokeir and Mousa, 2015 [47]	4	15	2	1	22
Strowitzki et al., 2012 [48]	4	15	1	1	21
Strowitzki et al., 2010 [49]	4	15	0	1	20
Taylor et al., 2017 [50]	4	14	1	1	20
Teixeira et al., 2017 [51]	4	15	3	1	23
Wayne et al., 2008 [52]	4	14	1	0	19
Wong et al., 2010 [53]	4	13	1	1	19
Zou et al., 2013 [54]	2	11	0	1	14

**Table 3:** The scores for open-label randomized clinical trials.

Author, Year [Ref]	TABO	Methods	Randomization	Results	Total Score
Ghahiri et al., 2012 [55]	3	11	0	1	15
Cheewadhanaraks et al., 2012 [56]	4	11	2	1	18
Ferrero et al., 2011 [57]	4	11	2	1	18
Gong et al., 2015 [58]	4	11	1	1	17
Kamencic and Thiel, 2008 [59]	4	13	0	1	18
Köhler et al., 2010 [60]	4	13	1	1	19
Strowitzki et al., 2010 [61]	4	15	1	1	21
Walch et al., 2009 [62]	3	13	1	1	18

TABO = Title/Abstract/Background and objectives; Score max. = 23 pt.

**Table 4:** Quality of randomized clinical trials in different medical fields.

Medical Field [Ref]	TABO	Random Sequence Generation	Allocation Concealment	Blinding Methods
Neurosurgery [12]	Good quality: most of the objectives			Poorly reported: 65.8%
Neuro-oncology [18]				Poorly reported: 70%
Respiratory physiotherapy post coronary bypass grafting [66]	Inadequate titles	Correctly reported: 51.28%	Insufficient details	Both patients and investigators: 7.69%
Nursing [67]		The type applied: 1.8%	Described: 0.3%	Specified: 5.9%
Nursing [68]		Good description: 3.8%		
Traditional Chinese nursing [69]		Described: 7.8%	Described: 1.4%	

TABO = Title/Abstract/Background and objectives.
